# Simulation of Phthisis by Disease of Pharynx and Nose

**Published:** 1907-11-16

**Authors:** 


### Laryngology and Rhinology.

### SIMULATION OF PHTHISIS BY DISEASE OF PHARYNX AND NOSE.

A considerable number of patients present themselves for examination and advice, complaining of symptoms which make them fear that they are consumptive, when their troubles are in reality entirely due to disease of the nose or throat. Such cases are a source of great anxiety to the medical man, for, although on examination of the chest no physical signs of disease can be detected, it is always difficult in the face of suspicious symptoms to state positively that no tuberculous disease of the lung is present. Thus, as a result of the very proper desire to treat every case of phthisis at the earliest possible stage, patients may be, and sometimes are, sent to sanatoria when, after all, they are not consumptive. In either direction an error has serious consequences.

The symptoms complained of are cough with purulent or muco-purulent expectoration, lack of appetite, loss of weight and strength, and general failure of health. If no definite signs of pulmonary tuberculosis can be discovered, it is well to remember that all these symptoms may be caused by nasal or pharyngeal disease; for this, if for no other reason, it is important that the medical adviser should be sufficiently skilful in the examination of these regions to recognise, at any rate, obvious disease of the nose and throat. It is a fact that at a large hospital for diseases of the chest in London these parts are, in a considerable proportion of cases, alone the seat of the disease which causes symptoms sufficiently alarming to bring the patients there for advice.

In children and adolescents this is especially the case with adenoid growths; these are not only a common cause of cough, but they also sometimes produce a marked degree of malnutrition, failure of growth, loss of flesh and anorexia. Bearing in mind the physiological action of the nose in cleansing, warming, and moistening the respired air, it is by no means improbable that nasal obstruction in young people is an important factor in the ætiology of pulmonary tuberculosis. When, however, the patient is not really consumptive, the removal of the adenoids, combined with general treatment, has a very gratifying result, for the patients increase rapidly in weight and in strength, and in many cases all fears of consumption are soon dispelled.

Other forms of nasal obstruction in older people have a similar effect. Any form of suppuration in the nose, such as atrophic rhinitis, or disease of the accessory sinuses, may have the same effect in a marked degree. The "latent" empyemata of the nasal sinuses are important from this point of view; the discharge may be so slight as to attract no attention except during exacerbations which the patient looks upon as "colds," to which he thinks he is unusually subject. Nevertheless the pus may produce by chronic intoxication an extraordinary degree of ill-health, which, with the cough and expectoration, closely simulates the symptoms of phthisis. During the quiescent stage no pus may be visible in the nose at any one examination, and the disease may readily escape detection; a little crust of dried secretion on the middle turbinal body is, however, usually to be seen, and should arouse suspicion. Chronic pharyngitis, particularly granular pharyngitis, is apt to cause cough and expectoration, and many patients, especially elderly women, come for the treatment of pulmonary disease in whom no other cause can be found to explain the symptoms. These are cases of considerable difficulty, for the severity of the symptoms is out of all proportion to the objective appearances; the characteristic pink lymphoid nodules and enlarged vessels are usually visible, the lateral bands of the pharynx (just behind the posterior faucial pillars) are often swollen, and the "lingual tonsil" is also frequently enlarged.

In cases of hæmoptysis, in which no signs of pulmonary phthisis are to be found, the question always arises as to whether the blood may not have come from some part of the upper respiratory tract. Unfortunately, in the majority of cases it proceeds from a focus in the lungs which has not, as yet, given rise to physical signs. Nevertheless hæmorrhage does take place from the upper parts of the respiratory tract, and it is very important that it should be looked for as soon as possible after it has occurred, in order that the diagnosis may be established; for if the bleeding spot can be found in the throat the patient will be spared an unnecessary sojourn in a sanatorium as well as much anxiety. More than one patient has been sent away for sanatorium treatment for bleeding from the throat. It is not difficult, with the use of care and cocaine, to avoid during the examination that coughing which is so dangerous in hæmoptysis. If the examination be delayed, it is frequently impossible to substantiate, or to exclude, with certainty the source of the hæmorrhage. The bleeding may come from any part of the upper respiratory tract; from an erosion or an enlarged vessel in the larynx, pharynx, tongue, or nose. Perhaps the most common sites are a shrivelled adenoid pad and the base of the tongue. In the latter situation enlarged veins are almost normal, and probably do not give rise to hæmorrhage; but in certain cases small round saccular varicosities may be seen here, and from these bleeding can undoubtedly occur. The bleeding may take place at any time and may be profuse, but usually it is only in the form of streaks or of a scanty brown expectoration on first rising in the morning; the blood is neither frothy nor intimately mixed with the sputum. After a genuine pulmonary hæmoptysis the sputum always remains somewhat tinged for a few hours, and only gradually loses its colour, whereas when the hæmorrhage comes from the throat the colouration ceases as soon as the bleeding stops. The patient's own impressions are of little value here, for he is often positive that he only hawked the blood from the throat, when it has undoubtedly proceeded from his lungs. A rusty-brown morning expectoration is very common in mouth-breathers. The gums are also a frequent source of small hæmorrhages, and should always be examined in these cases.

